# EXPLORING THE EFFECT OF EXOSOMES AND RADIATION ON GLIOBLASTOMA CANCER CELLS

**DOI:** 10.1093/geroni/igad104.3124

**Published:** 2023-12-21

**Authors:** Hannah Hausman, Linda Azizi, Frank Pajonk

**Affiliations:** St. Catherine University, St. Paul, Minnesota, United States; University of California, Los Angeles, Los Angeles, California, United States; University of California, Los Angeles, Los Angeles, California, United States

## Abstract

Glioblastoma is the most common and aggressive type of brain cancer, with higher incidences in aging adults over 75 and little to no long-term survival or cure. The median survival time under the current standard of care, surgical resection and chemoradiation, is 15-18 months. Glioblastoma tumors consist of multiple cell types, including mature brain cells in cell cycle arrest and cancer stem cells (CSCs) that replenish cancer cell populations. However, the cancerous non-stem cells are induced into a cell fate called glioma-initiating cells in response to radiation, where they begin to express stem-like phenotypes. Glioma-initiating cells proliferate at an aggressive rate and are the reason for cancer recurrence, making them a necessary target for new and more effective therapeutics. This research is studying the effect of exposure to exosomes, extracellular vesicles that carry proteins and RNA, on the stem-like phenotype expression of irradiated non-stem cells. Irradiated glioblastoma cells were exposed to varying concentrations of exosomes isolated from glioblastoma CSCs in vitro. The results showed a decrease in ZsGreen expression, a marker for stem-like phenotypes, as exosome concentration increased. Exposure of glioblastoma cancer cells to exosomes post-irradiation decreased the rate of transition to glioma-initiating cells. This suggests that CSC exosomes may signal that stem cells are present, decreasing the need for the formation of glioma-initiating cells. Further research is necessary to better understand the role of exosomes in glioblastoma tumors, however, this result may suggest exosomes as a potential way to delay or reduce the aggressive nature of glioblastoma cancer recurrence.

